# Streptococcal and *Staphylococcus aureus* prosthetic joint infections: are they really different?

**DOI:** 10.1186/s12879-022-07532-x

**Published:** 2022-06-17

**Authors:** Yousra Kherabi, Valérie Zeller, Younes Kerroumi, Vanina Meyssonnier, Beate Heym, Olivier Lidove, Simon Marmor

**Affiliations:** 1 Centre de Référence des Infections Ostéo-Articulaires Complexes, Groupe Hospitalier Diaconesses-Croix Saint-Simon, 125, rue d’Avron, 75020 Paris, France; 2 Service de Médecine Interne et Infectiologie, Groupe Hospitalier Diaconesses-Croix Saint-Simon, 125, rue d’Avron, 75020 Paris, France; 3 Laboratoire des Centres de Santé et Hôpitaux d’Île-de-France, Groupe Hospitalier Diaconesses-Croix Saint-Simon, 125, rue d’Avron, 75020 Paris, France; 4 Service de Chirurgie Osseuse et Traumatologique, Groupe Hospitalier Diaconesses-Croix Saint-Simon, 125, rue d’Avron, 75020 Paris, France

**Keywords:** Prosthetic joint infection, Methicillin-susceptible *Staphylococcus aureus*, *Streptococcus* spp

## Abstract

**Background:**

Staphylococci and streptococci are the most frequent pathogens isolated from prosthetic joint infections (PJIs). The aim of this study was to analyze the outcome of streptococcal and methicillin-susceptible *Staphylococcus aureus* (MSSA) PJIs.

**Methods:**

All monomicrobial streptococcal and MSSA PJIs managed in a French Referral Center (2010–2017) were sampled from the prospective PJIs cohort study. The primary outcome of interest was the cumulative reinfection-free survival at a 2-year follow-up.

**Results:**

Two hundred and nine patients with 91 streptococcal and 132 staphylococcal infections were analyzed. Patients with streptococcal PJI were older, and infection was more frequently hematogenous. Reinfection-free survival rates at 2-years after all treatment strategies were higher for patients with streptococcal PJI (91% vs 81%; *P* = .012), but differed according to the strategy. After exchange arthroplasty, no outcome differences were observed (89% vs 93%; *P* = .878); after debridement, antibiotics and implant retention (DAIR), the reinfection-free survival rate was higher for patients with streptococcal PJI (87% vs 60%; *P* = .062). For patients managed with prolonged suppressive antibiotic therapy (SAT) alone, those with streptococcal PJIs had a 100% infection-free survival (100% vs 31%; *P* < .0001).

**Conclusions:**

Reinfection-free survival after DAIR and SAT was better for patients with streptococcal than those with MSSA PJIs. No difference was observed after prosthesis exchange.

**Supplementary Information:**

The online version contains supplementary material available at 10.1186/s12879-022-07532-x.

## Background

Although rare, prosthetic joint infection (PJI) is a severe complication of total hip and knee arthroplasties, with high morbidity and medical costs [1–4]. Staphylococci are the main pathogens isolated from PJIs, with *Staphylococcus aureus* being the most frequent species, responsible for 19–29% of PJIs, and streptococci, being the second most frequent microorganism with Gram negative bacilli, found in 9–16% of PJIs [5–7]. *Streptococcus spp.* are the most frequent microorganism isolated in hematogenous acquired PJIs [5–7].

It is now well-known that *S. aureus* virulence factors provide enhanced adherence capacity to implants [8]. Once infection is established, *S. aureus* is able to form biofilm, modify its metabolism, grow in small colonies and survive in this microenvironment [9, 10]. Streptococci can also form biofilm [11, 12], but less is known about it than that of *S. aureus* [13]. Biofilm active antibiotics are not recommended to treat streptococcal infections [1, 14], but the use of rifampicin-combination therapy is not rare and remains controversial [15, 16]. Streptococci usually have very low minimum inhibitory concentration (MIC). Indeed, amoxicillin MIC distribution ranges for *S. agalactiae* are 0.016–0.125 mg/L, i.e. much lower than *S. aureus* (oxacillin MIC distribution for methicillin-susceptible *S. aureus* 0.06–2 mg/L) [17]. Clinical experience suggests that streptococcal PJI outcomes are better than those with *S. aureus* [13, 18].

Numerous studies on *S. aureus* PJI treated with debridement, antibiotics and implant retention (DAIR) have been published over the last decade [19–21]. Only a few large retrospective studies addressed streptococcal PJIs [15, 16, 22, 23]. Their characteristics and outcomes remain less known. Lora-Tamayo et al. published the largest study including 462 DAIR-treated PJIs [15]. Their outcomes did not appear to be better than those of patients with *S. aureus* PJIs, as 42% of the patients experienced failure. Another multicentric study including 70 streptococcal PJIs found that DAIR and *S. agalactiae* were associated with a higher risk of failure [16]. At 2-year follow-up, 51% of their patients treated with DAIR had relapsed. However, the authors of two small comparative studies on DAIR-treated patients concluded that streptococcal PJI outcomes were better than those of *S. aureus* PJIs [24, 25]. A large study on patients treated with DAIR for late acute PJI also indicated better results for streptococcal than MSSA infections (respective failure rates 37% vs 55%) [26]. For acute hematogenous PJIs, *S. aureus* was identified as a negative prognostic factor compared to other etiologies [18].

The lack of studies analyzing specifically streptococcal and *S. aureus* PJI outcomes and treatments, and the high rates of treatment failure reported for streptococcal PJIs treated with DAIR [15, 16], led us to question outcomes between the two microorganisms and according to the therapeutic strategy applied.

We performed an observational cohort study analyzing the outcomes of streptococcal and methicillin-susceptible *S. aureus* (MSSA) PJIs, their epidemiological and clinical characteristics, and their therapeutic strategies.

## Patients and methods

### Study design

This cohort study was conducted in a French National Referral Center for Bone-and-Joint Infections (BJI) [27]. All patients admitted to our Referral Center for PJIs are registered in the prospective PJI cohort (NCT 01963520, NCT 02801253). Epidemiological, clinical, microbiological, therapeutic (surgery and antibiotic therapy), adverse event and outcome data of each patient are entered prospectively. The primary outcome of the cohort is the 2-year-reinfection-free survival.

All patients, 18 years of age or older, treated from January 2010 to July 2017 for a streptococcal (any species) or MSSA hip and/or knee PJI(s), with a curative strategy or prolonged suppressive antibiotic therapy (SAT), were included. Polymicrobial, methicillin-resistant *S. aureus* (MRSA) and enterococcal PJIs were not included.

A retrospective analysis of those prospectively collected data was performed.

### PJI definitions and classification

PJI was defined as the isolation of the same microorganism from ≥ 2 cultures of preoperative joint-fluid and/or intraoperative tissue specimens plus at least 1 of the following criteria: a sinus tract communicating with the prosthesis, local inflammatory signs, C-reactive protein > 5 mg/L and/or radiological findings (ie, periosteal bone formation, subchondral osteolysis) [28].

PJIs lasting for < 3 weeks were defined as acute, and the others as chronic [1, 6]. Early-postoperative infection was defined as surgical site pain, redness with or without drainage, associated or not with fever, occurring within 30 days after joint arthroplasty. Late-chronic infection was defined as progressive pain, joint dysfunction with or without a fistula, occurring more than 30 days after joint arthroplasty. A hematogenous infection was defined as occurring after a symptom-free interval of at least 30-days post-surgery, with sudden onset of pain, joint dysfunction with or without fever, and/or chills [7].

### Microbiological diagnosis

Preoperative joint aspiration was performed for all patients, except for patients with an early postoperative PJI operated right away after admission. Joint aspiration was done in the department of radiology under strict sterile conditions [7]. During surgery at least 3 intraoperative samples of bone and/or synovium that appeared inflamed were collected before starting antibiotics. Tissue or bone specimens were disrupted by vigorous crushing in sterile mortars with sterile diluents. For cultures, aliquots of the resulting suspensions and/or synovial fluid were inoculated onto PolyViteX (PVX) chocolate agar (incubated under 5% CO_2_) and anaerobic Columbia agar plates (bioMérieux, Marcy-l’Étoile, France) and into aerobic (Hemoline, bioMérieux), and into anaerobic enrichment broths (Schaedler broth, bioMérieux). Cultures were incubated for 10 days for aerobic and 14 days for anaerobic cultures. On day 10 or 14, or earlier if bacterial growth was visible, broths were subcultured on PVX chocolate agar and anaerobic Columbia agar plates, and incubated at 37 °C for 48 h [7].

Bacteria were identified to species with the rapid ID 32A kit (bioMérieux) and, since January 2012, by mass spectrometry (MALDI biotyper, Bruker Dalton, Bremen, Germany). Antibiotic-susceptibility testing used the standard disk-diffusion method, according to the recommendations of the French Society of Microbiology [29]. Molecular biology methods (Polymerase Chain Reaction) were not used to identify microorganism.

### Therapeutic strategies

**Fig. 1:**
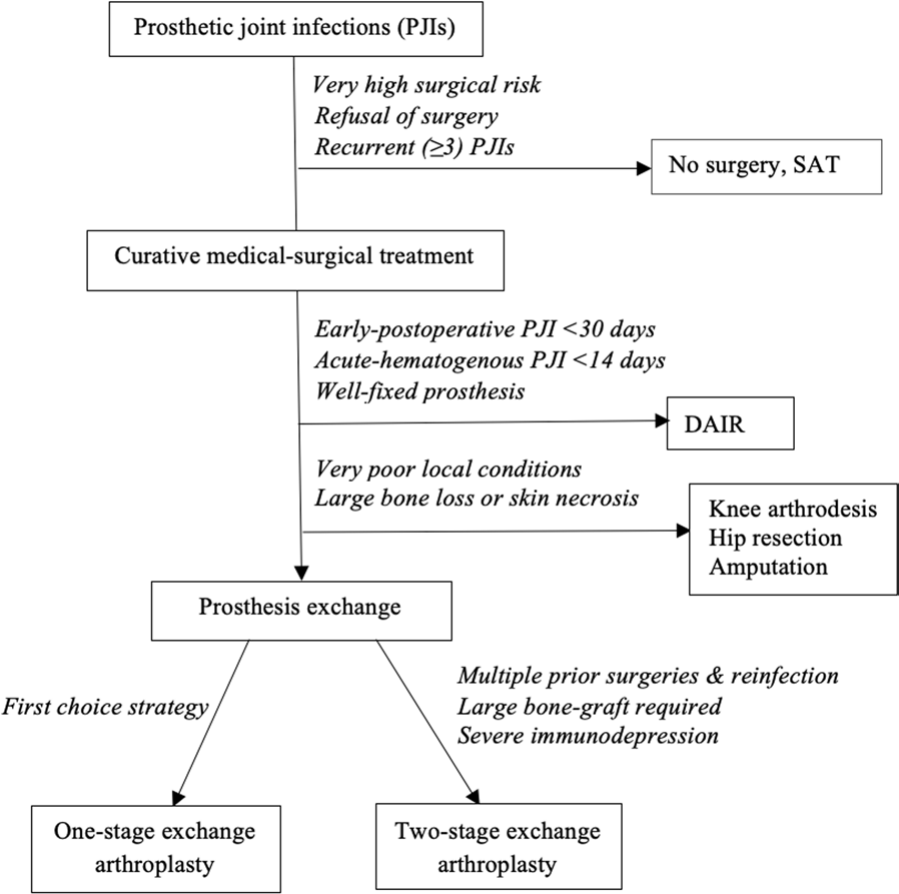
Surgical and medical strategies for prosthetic joint infections (PJIs) treatment. *PJIs* prosthetic joint infections, *DAIR* debridement, antibiotics and implant retention, *SAT* prolonged suppressive antibiotic therapy

Curative surgical treatment included DAIR for early postoperative infections (developing within 30 days postsurgery) and acute hematogenous PJIs lasting < 2 weeks without prosthesis loosening. Otherwise, complete prosthesis exchange arthroplasty was done, most often one-stage exchange [30]. Two-stage exchange was performed if several negative prognostic factors were associated (irradiated bone, severe immunosuppression, large bone graft required …) (Fig. 1). Five MSSA-PJI patients underwent definitive prosthesis removal.

Antibiotic therapy for acute PJI treated with DAIR lasted 6 weeks: 2–4 weeks intravenous, followed by a 2–4-week oral regimen. For chronic PJIs treated with prosthesis exchange, 12-week antibiotic therapy comprised 2–4 weeks of intravenous therapy. It was shortened to 6 weeks in January 2017.

Pathogen-specific antibiotic therapies were based on international and French recommendations [1, 31] and our local procedures, detailed in a recent article [32]. First choice treatment for streptococcal infection in non-allergic patients was amoxicillin, given IV at high doses and prolonged infusion. Amoxicillin was also used in these patients for the oral switch, 2–3 g, thrice daily. Before January 2013, rifampicin was used to treat streptococcal and staphylococcal PJIs. Since then, rifampicin in combination has only been prescribed for staphylococcal PJIs [32].

For MSSA PJI, continuous IV cefazolin infusion combined to IV rifampicin (600–900 mg, twice a day) was the first choice treatment followed by oral therapy with levofloxacin (750 mg/day to 500 mg twice a day) and rifampicin (600–900 mg, twice a day) [32, 33].

SAT was not prescribed for patients who underwent curative surgical treatment (DAIR, prosthesis exchange or definitive prosthesis removal). This specific treatment option was applied right away to patients at high surgical risk, recurrent PJIs (> 2 PJIs), or who refused surgery (Fig. 1). Initial antibiotic therapy was administered intravenous for 7–10 days and then continued orally. SAT was given for at least 3 months and up to several years, depending on patient’s evolution and antibiotic tolerance. Drug dosage for oral SAT was 1 g thrice a day for amoxicillin, cloxacillin and cephalexin, and 600 mg thrice a day for clindamycin. SAT was discontinued when a serious adverse event occurred as defined by the Food and Drug Administration [34].

All treatment options were discussed in multidisciplinary meetings, requiring the presence of at least an orthopedic surgeon, a microbiologist and an infectious disease specialist.

### Outcome measures

Patients were followed for at least 2 years. The following events were recorded: reinfection, either relapse with the same pathogen or new infection with a different microorganism, and death from any cause. No patient was lost-to-follow-up at 2 years.

### Statistical analyses

The primary outcome of interest was the cumulative reinfection-free survival at a 2-year follow-up. Qualitative variables were expressed as number (%) and compared using χ^2^ test. Quantitative variables were first assessed for normality, expressed as median [interquartile range (IQR)], and then compared with the non-parametric Mann–Whitney *U*-test. The reinfection-free–survival rate was analyzed using the Kaplan–Meier method and expressed as the mean ± standard deviation. The Mantel–Cox log-rank test was used to calculate survival-distribution difference. A *P* value < 0.05 was considered significant. All statistical tests were computed with SPSS.20 software.

### Ethics approval

Written informed consent was obtained from all patients and the cohort was approved by the Ethics Committee of Île-de-France (N° PP 14–034).

## Results

### Patient and PJI characteristics

**Fig. 2:**
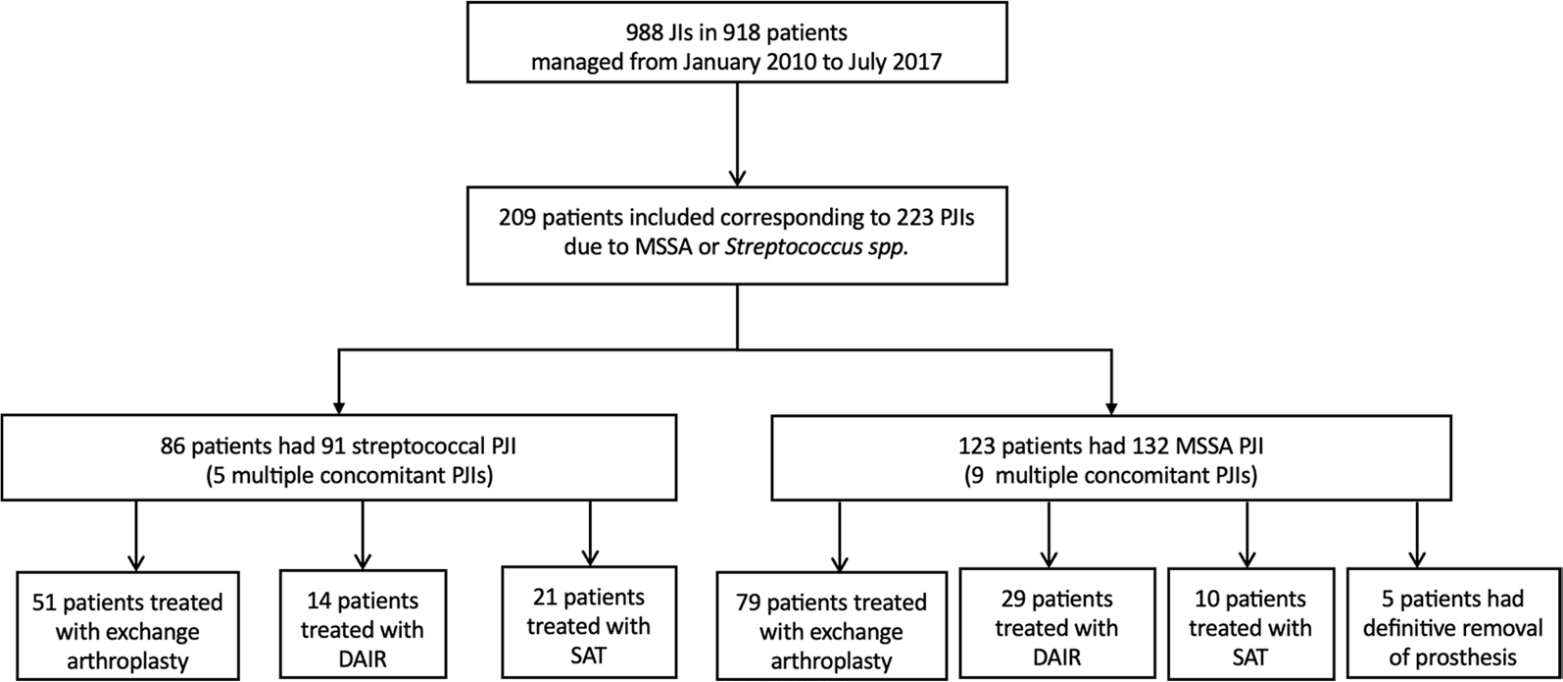
Flowchart of the entire population. *JIs* joint infections, *PJI* prosthetic joint infection, *MSSA* methicillin-susceptible *Staphylococcus aureus*, *DAIR* debridement, antibiotics and implant retention, *SAT* prolonged suppressive antibiotic therapy

During the 7.5-year study period, 988 PJIs in 918 patients were managed in our center. Two hundred and nine patients, 86 with 91 streptococcal and 123 with 132 MSSA PJIs, for a total of 223 PJIs, were included (Fig. 2). Five patients with streptococcal and 9 with MSSA PJIs had multiple concomitant prosthetic hip or knee infections.

Patients’ description and PJIs characteristics are reported in Table 1.

**Table 1:** Baseline characteristics of patients with Streptococcal or MSSA PJIs

Characteristic	Streptococcal PJIs patients (n = 86)	MSSA PJIs patients (n = 123)	*P*
Age (years)	76 [68–84]^a^	73 [65–80]	0.030
Sex (male/female)	41 (48) / 45 (52)	63 (51) / 60 (49)	0.674
ASA score ≥ 3	41 (48)	64 (52)	0.575
Body mass index (kg/m.^2^)	28 [24-30]	27 [23-30]	0.318
Atrial fibrillation	7 (20)	19 (15)	0.457
Cardiovascular diseases	34 (40)	34 (28)	0.072
Hypertension	57 (66)	76 (62)	0.466
Immunosuppressive therapy	2 (2)	7 (6)	0.315
Rheumatological inflammatory diseases	4 (5)	6 (5)	1.000
Diabetes	10 (11)	25 (20)	0.132
Chronic dermatosis	8 (9)	6 (5)	0.262
Malignancy within the last 5 years	8 (9)	3 (2)	0.054
Renal insufficiency.^b^	4 (5)	6 (5)	1.000
Cirrhosis	1 (1)	3 (2)	0.646
Prosthetic knee infection	31 (36)	55 (45)	0.253
Prosthetic hip infection	55 (64)	68 (55)	0.253
Multiple concomitant PJIs	5 (6)	7 (6)	1.000
Bilateral knee PJIs	2 (2)	4 (3)	1.000
Bilateral hip PJIs	3 (3)	3 (2)	1.000
Prior PJI-treatment and failure	14 (16)	49 (40)	< 0.0001
Acute PJIs (lasting < 3 weeks at admission)	42 (49)	45 (37)	0.088
PJI duration before surgery in our Center (days)	6 [2–12]	7 [4–13]	0.356
Postoperative PJI	0	17 (38)	< 0.0001
Hematogenous PJI	42 (100)	24 (53)	< 0.0001
Unknown	0	4 (9)	0.117
Chronic PJIs (lasting > 3 weeks at admission)	44 (51)	78 (63)	0.088
PJI duration before surgery in our Center (days)	200 [97–355]	171 [113–330]	0.695
Postoperative PJIs	2 (5)	30 (38)	0.001
Hematogenous PJIs	33 (75)	34 (44)	0.001
Unknown	9 (20)	14 (18)	0.811

*ASA* American Society of Anesthesiologists, *DAIR* debridement, antibiotics and implant retention, *MSSA* methicillin-susceptible *Staphylococcus aureus*, *PJI* prosthetic joint infection

^a^Results are expressed as number (%) or median [interquartile range] for continuous variables

^b^eGFR < 30 mL/min/1.73 m^2^

Patients with streptococcal PJI were significantly older than those with MSSA PJI, had cancer more frequently within the last 5 years and their PJIs were often hematogenously acquired (88% vs 46%; *P* < 0.0001). Patients with MSSA experienced more prior PJI treatment failures in another center (40% vs 16%; *P* < 0.0001).

### Microbiology

Streptococcal species isolated from these 86 patients’ PJIs are detailed in Additional file 1: Table S1. The most frequently isolated species was *Streptococcus agalactiae* (33%), *Streptococcus dysgalactiae* (20%) and *Streptococcus mitis/oralis* (16%).

### Surgical and medical treatments

Details on treatment strategies and antibiotic therapy are given in Table 2. Rates of curative surgical strategies, ie, exchange arthroplasty and DAIR, did not differ between patients with streptococcal and those with MSSA PJIs.

**Table 2:** Treatment strategies for patients with Streptococcal or MSSA PJIs

Treatment strategy	Streptococcal PJIs (n = 86)	MSSA PJIs (n = 123)	*P*
Exchange arthroplasty	51 (59)^a^	79 (64)	0.389
Time (days) to prosthesis exchange	22 [5–196]	92 [12–257]	0.062
One-stage exchange	50 (98)	74 (94)	0.670
Two-stage exchange	1 (2)	5 (6)	0.404
DAIR	14 (16)	29 (24)	0.306
Time to DAIR (days)	5 [2–9]	8 [4–12]	0.042
Mobile device exchange	8 (57)	14 (48)	0.51
Definitive prosthesis removal	0	5	0.079
Antibiotics combined with surgery			
Treatment duration (days)	87 [85–92]	87 [64–92]	0.444
IV duration (days)	32 [29–36]	33 [29–44]	0.265
Beta-lactams > 14 days	65 (76)	85 (69)	< 0.0001
Rifampicin > 14 days	29 (35)	88 (73)	< 0.0001
Prolonged suppressive antibiotic therapy	21 (24)	10 (8)	0.001
Initial IV antibiotics	16 (55)	8 (80)	1.000
IV duration (days)	9 [4-23]	16 [11–27]	0.071
Duration (days)	405 [375–741]	366 [136–640]	0.016

*DAIR* debridement, antibiotics and implant retention, *IV* intravenous, *MSSA* methicillin-susceptible *Staphylococcus aureus*, *PJI* prosthetic joint infection

^a^Results are expressed as number (%) or median [interquartile range] for continuous variables

One-stage exchange arthroplasty was by far the most frequent operation. Among these 124 patients, prior treatment failure was significantly more frequent in patients with MSSA PJIs (35/74; 47% vs 9/50; 18%, *P* < 0.0001). Furthermore, 8 patients with streptococcal and 9 with MSSA acute PJIs of a loosened prosthesis or hematogenous infection lasting > 2 weeks underwent exchange arthroplasty: 16 underwent one-stage and one MSSA PJI-patient underwent two-stage exchange arthroplasty. All but 2, received preoperative antibiotic therapy for a median of 7 [2–18] days before exchange arthroplasty.

Six patients with prosthetic hip infection underwent two-stage arthroplasty. No antibiotic-loaded cement was used.

Moreover, 5 patients with MSSA PJIs had their prostheses definitively removed (2 hip-resection arthroplasties and 3 knee arthrodeses).

SAT was prescribed significantly more often to treat streptococcal PJI patients (24% vs 8%, *P* = 0.001). None of the SAT-patients underwent surgery for their ongoing PJI. All streptococcal PJI-patients received first-line antibiotic therapy with amoxicillin, intravenously and then orally, except for 5 patients who had oral amoxicillin right away. All SAT-patients with MSSA PJIs received intravenous cefazolin or oxacillin, followed by oral cloxacillin (n = 8), clindamycin (n = 2) or cefalexin (n = 2).

### Outcomes

**Fig. 3:**
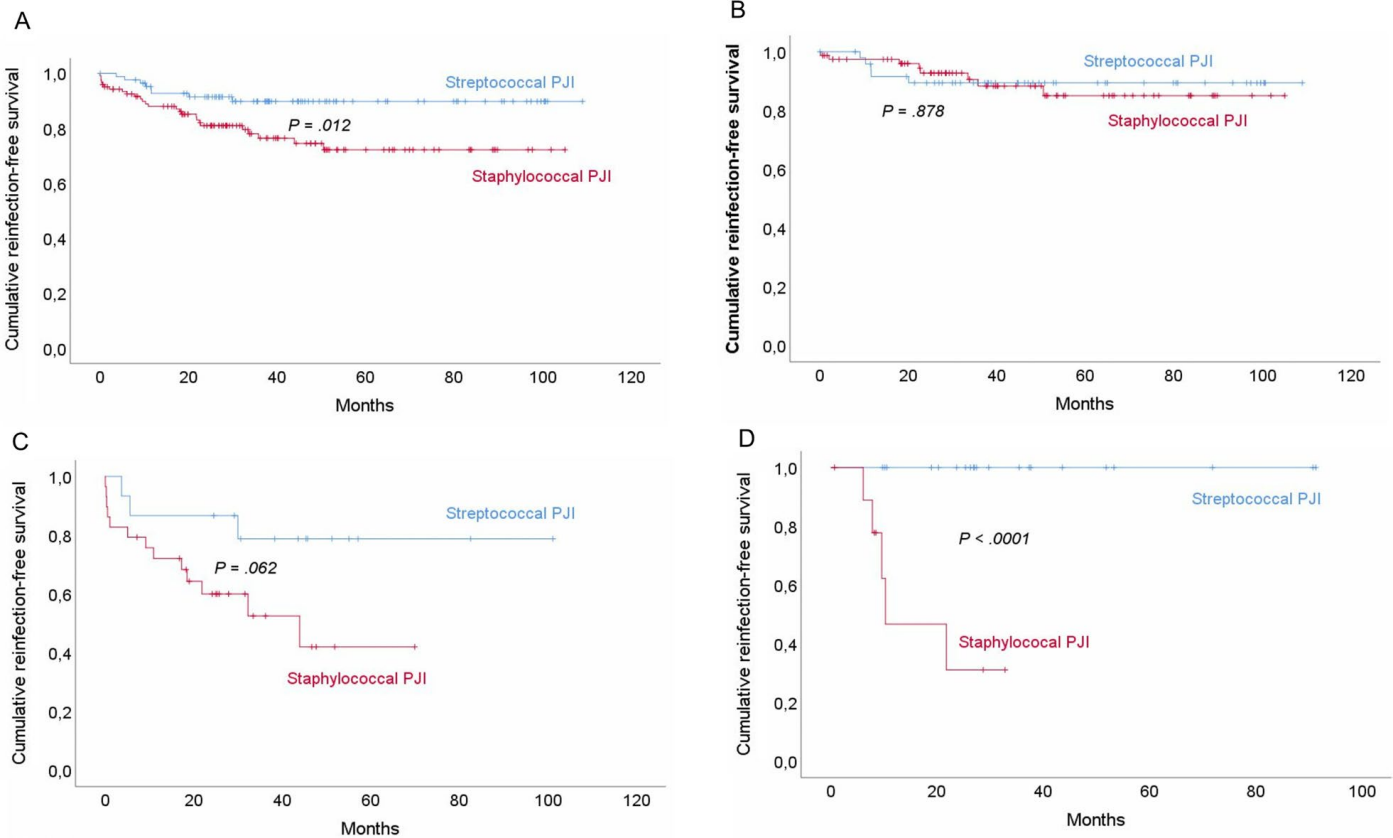
Cumulative reinfection-free survival rates of patients with streptococcal or methicillin-susceptible *S. aureus* prosthetic joint infection (PJIs). **A** Entire population, all strategies (n = 209; 86 streptococcal and 123 MSSA patients). **B** After prosthesis exchange (n = 130; 51 streptococcal and 79 MSSA patients). **C** After debridement and implant retention (DAIR) (n = 43; 14 streptococcal and 29 MSSA patients. **D** After prolonged suppressive antibiotic therapy (SAT) (n = 31; 21 streptococcal and 10 MSSA patients)

At 2 years (Table 3), all therapeutic strategies combined, reinfection-free survival was higher for patients with streptococcal than MSSA PJIs (Fig. 3A). Eight patients with streptococcal PJIs experienced reinfections (5 relapses, 3 new-pathogen PJIs) compared to 26 patients with MSSA PJIs (15 relapses, 11 new-pathogen PJIs).

**Table 3:** Reinfection-free survival rates for patients with Streptococcal or MSSA PJIs after 2 years of follow-up according to treatment

Treatment	Streptococcal PJIs	Staphylococcal PJIs	*P* Log-rank (Mantel–Cox)
All strategies	91.4 ± 0.03^a^	81 ± 0.03	0.012
Prosthesis exchange	89.4 ± 0.04	92.7 ± 0.03	0.878
DAIR	86.7 ± 0.08	60 ± 0.09	0.062
SAT	100	31.1 ± 0.17	< 0.0001

*DAIR* debridement, antibiotics and implant retention, *MSSA* methicillin-susceptible *Staphylococcus aureus*, *PJIs* prosthetic joint infections, *SAT* prolonged suppressive antibiotic therapy

^a^Results are expressed as mean percentage ± standard deviation

Between patients with streptococcal and those with MSSA PJIs, differences were observed according to the medical–surgical strategy. After exchange arthroplasty, no difference in outcome was noted and reinfection-free survival was ~ 90% for streptococcal and MSSA PJI-patients (Fig. 3B). *Streptococcus dysgalactiae* was responsible for both streptococcal knee-arthroplasty relapses. The 2 patients with MSSA PJIs who relapsed were 78-year-old males. New infections occurred in 3 patients with streptococcal PJIs and 6 with MSSA PJIs.

However, after DAIR, reinfection-free survival was higher for patients with streptococcal PJIs (Fig. 3C). Three relapses (21%) occurred with *S. agalactiae*, *S. dysgalactiae* or *S. salivarius*, without any new-pathogen infections. Patients with MSSA PJIs experienced 8 relapses (28%) and 5 new infections.

For patients with streptococcal PJIs treated with prosthesis exchange or DAIR, no outcome differences were observed between those who were on rifampicin or not (2 relapses among 30 treated with combination therapy vs 3 among 35 given beta-lactam monotherapy) (*P* = 1.000).

Among patients treated with SAT, those with streptococcal PJIs (Fig. 3D) had better outcomes, and none had treatment failures. Only 1 patient stopped amoxicillin after 10 months, because of *Clostridioides difficile* colitis; he died 9 months later of metastatic prostate neoplasia. Five (42%) patients with MSSA PJIs relapsed: 1 following treatment discontinuation after 1 year of cloxacillin relayed by cephalexin and four others while taking oral cloxacillin.

**Fig. 4:**
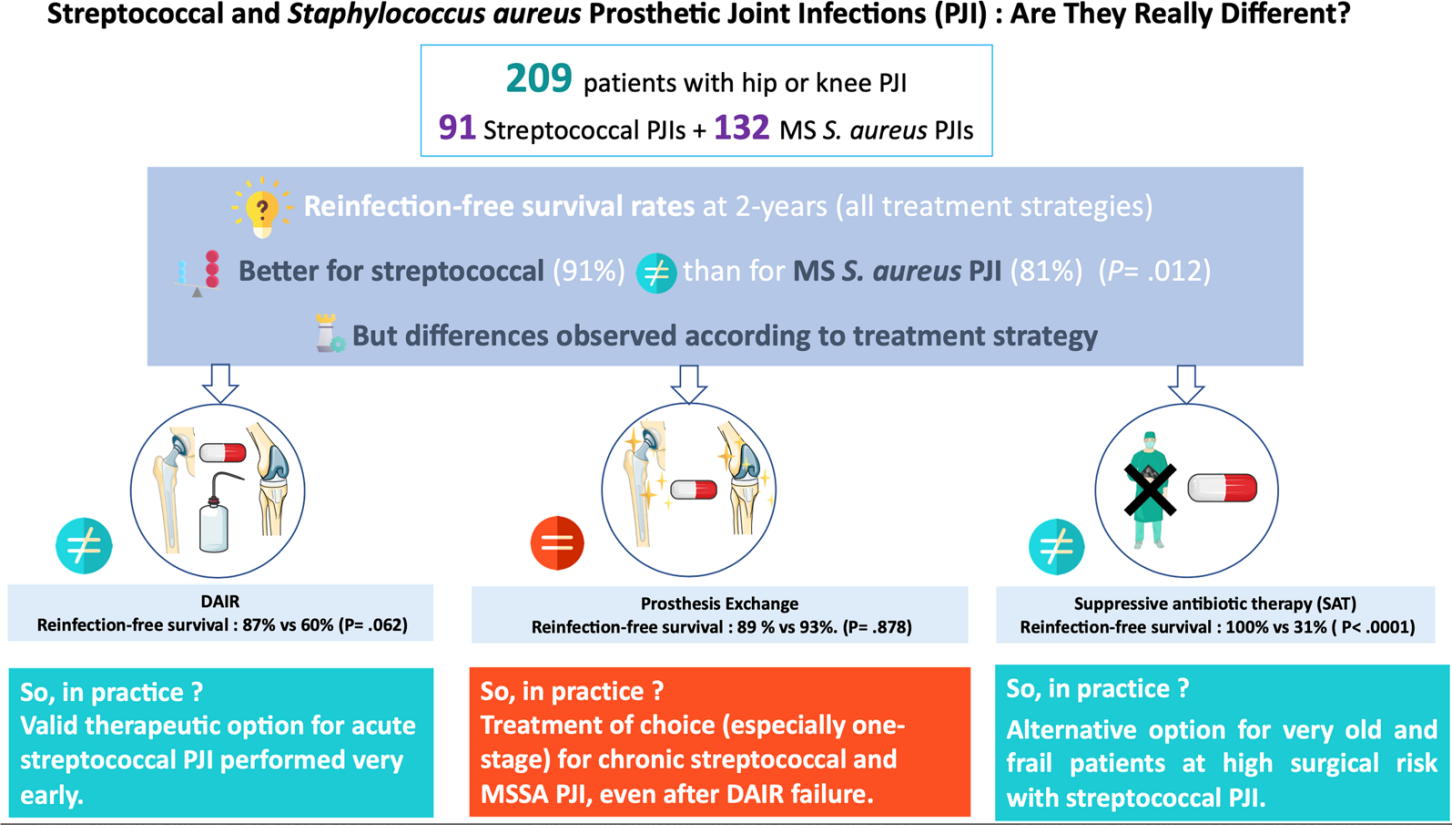
Research summary. *JIs* joint infections, *PJI* prosthetic joint infection, *MSSA* methicillin-susceptible *Staphylococcus aureus*, *DAIR* debridement, antibiotics and implant retention, *SAT* prolonged suppressive antibiotic therapy

Mains results are summarized in an overview figure (Fig. 4).

## Discussion

The results of our large observational cohort study on outcomes and characteristics of streptococcal and MSSA PJIs showed that patients with streptococcal PJIs were older, more frequently had cancer, and their hematogenous infection rate was higher. These findings are consistent with previous observations [15, 22]. Prior PJI-treatment failure before admission to our center was significantly more frequent for patients with MSSA than streptococcal PJIs.

Our analysis showed also that streptococcal PJIs had better outcomes than MSSA for all therapeutic strategies combined, with differences observed according to the medical–surgical strategy. Notably, no difference was observed after prosthesis exchange. Although outcomes were better in patients treated with DAIR for their streptococcal PJIs, the difference was not significant, probably because the study lacked power. The median time to DAIR was significantly shorter and the rate of mobile components exchange was higher in patients with streptococcal PJI, but the difference wasn’t significant. These results could partly explain the better outcome of streptococcal PJI after DAIR.

*S. aureus* was previously identified as a risk factor for DAIR failure [26, 35, 36]. Its notable biofilm-formation capacity, a very efficient tool for bacterial survival, its longer interval before DAIR, and lower antibiotic susceptibility can explain the higher treatment-failure rate [17]. As Wouthuyzen-Bakker et al. [26] emphasized, an unrecognized, chronic PJI with a sudden clinical onset could be an additional reason for treatment failure in some patients. More than one-quarter of our patients with MSSA PJIs treated with DAIR relapsed, but reinfection was also attributed to new-pathogen PJIs, not observed in the streptococcal group. Since the numbers of patients treated with DAIR either for streptococcal or MSSA PJIs were small, conclusions should be drawn cautiously. Indeed, to date, DAIR remains, in our center, the treatment of choice for acute MSSA PJIs evolving for less than 2 weeks and without prosthesis loosening. Nevertheless, these results suggest a difference in populations and PJI types between these pathogens, with the MSSA PJI patients having a higher risk of reinfection. To improve patient care, the cohort patients that benefit from DAIR needs to be further characterized.

These crucial points of the pathophysiology of foreign-body infection could also explain the good and similar outcomes of our patients with streptococcal or MSSA PJIs treated with prosthesis exchange. The strength and originality of our study was to include many patients treated with exchange arthroplasty, especially one-stage exchanges, which had not been addressed previously. Exchange arthroplasty removes the entire foreign body containing the bacteria embedded in biofilm. In our experience, one-stage exchange arthroplasty has a very low relapse rate (≤ 5%) in chronic prosthetic hip infections [30], even MSSA PJIs, as shown herein. Interestingly, more MSSA-patients had underwent prior PJI management before admission to our center (40% vs 16%), but their outcome was not different from streptococcal PJI patients, and the number of relapses was very low (2.5%). One-stage arthroplasty is a valid and advantageous option for chronic PJIs. In their multicenter study on 70 streptococcal PJIs, Mahieu et al. also obtained good results with one- and two-stage arthroplasty, without any relapses [16]. However, the question remains whether one-stage exchange arthroplasty should be used as first-line treatment for acute hematogenous infection of a loosened prosthesis or subacute PJIs lasting > 2 weeks with a high bacterial load. In those settings, outcomes after exchange arthroplasty may be less favorable than for chronic PJIs [37, 38] and can lead to septic shock. The rationale behind why we start antibiotics preoperatively is to reduce the bacterial load and local inflammation. However, our findings have to be confirmed by future studies.

Another important and unresolved question is the benefit of rifampicin against streptococcal PJIs. During the first 3 years of this cohort, patients received combination therapy with rifampicin before being stopped because of the absence of data supporting its use and the occurrence of rifampicin-induced adverse events in one-third of patients (data not shown) [15, 39]. Notably, outcomes of patients with streptococcal PJI treated with or without rifampicin did not differ. Successful complete removal of the implants during exchange arthroplasty is certainly an important argument not to use rifampicin. Based on their large study, Lora-Tamayo et al. concluded that rifampicin-combination therapy for streptococcal PJIs treated with DAIR could improve the outcome, but has to be confirmed in further studies [15]. However, according to Mahieu et al.’s multicenter study [16], rifampicin-combination therapy did not achieve better outcomes.

Finally, streptococcal PJI patients treated with SAT had better outcomes than those with MSSA infections. This therapeutic option was prescribed more frequently to streptococcal PJI patients, especially those very old and frail, or with relapsing PJIs. These patients did not undergo surgery. Amoxicillin having excellent activity against *Streptococcus* spp. and high bioavailability yielded very good outcomes with good tolerance. No relapses occurred and only one patient stopped amoxicillin because of *Clostridioides difficile* colitis. Although this strategy avoids surgery and achieves good infection control, it should be reserved for patients at high surgical risk or those with frequent, recurrent infections. On the other hand, five MSSA PJI patients relapsed, four while taking oral cloxacillin. Lower *S. aureus* antibiotic susceptibility and cloxacillin bioavailability could explain the difference observed. American guidelines recommend cephalexin, dicloxacillin or clindamycin for SAT [1].

Our study has several limitations. First, it was a monocenter study conducted in a highly specialized referral center for treatment of complex BJI, thereby limiting the generalizability of our findings and carrying a risk for selection bias. For example, more MSSA PJI-patients had prior management of their PJI before admission to our center. This may have had an impact on our results. Moreover, as already mentioned in the methodology section, there were different antibiotic treatment protocols during the study period. The number of DAIR- or SAT-treated patients was small. Unlike the MSSA group, several streptococcal species were included, resulting in heterogeneity of the population.

In conclusion, we described a large cohort study analyzing streptococcal and MSSA PJIs. DAIR obtained a better reinfection-free survival rate for patients with streptococcal than MSSA PJIs, making it a valid therapeutic option for acute streptococcal PJI, if initiated very early. Outcomes after one-stage exchange arthroplasty were comparable with low relapse rates, highlighting the crucial role of this treatment strategy for chronic PJIs, even after DAIR failure. SAT is an alternative option for very old and frail patients at high surgical risk who develop streptococcal PJIs. The microorganism type, in this case *S. aureus* or *Streptococcus spp.*, is one of several important factors guiding medico-surgical strategy decision, in addition to infection duration, underlying comorbidities, functional state and surgical procedure complexity.

## Supplementary Information

**Additional file 1: Table S1.** Streptococcal Species Isolated from 86 Patients’ PJIs.

## Data Availability

The datasets used and/or analysed during the current study are available from the corresponding author on reasonable request.
