# Supplementary material for: Streptococcal and Staphylococcus aureus prosthetic joint infections: are they really different?
Source: BMC Infect Dis. 2022 Jun 17;22:555. doi: 10.1186/s12879-022-07532-x (PMC9206280; doi:10.1186/s12879-022-07532-x)
Supplement: Supplementary file 1 — Additional file 1: Table S1. Streptococcal Species Isolated from 86 Patients’ PJIs. [file 12879_2022_7532_MOESM1_ESM.docx]

**Additional material**

**Table S1. Streptococcal Species Isolated from 86 Patients’ PJIs**

| Species | Patients, n (%) |
| --- | --- |
| *S. agalactiae* | 28 (33) |
| *S. dysgalactiae* | 17 (20) |
| *S. mitis/oralis* | 14 (16) |
| *S. milleri (S. anginosus, S. constellatus, S. intermedius*) | 7 (8) |
| *S. gallolyticus* | 4 (5) |
| *S. pneumoniae* | 2 (2) |
| Others | 14 (16) |

Abbreviation: PJIs, prosthetic joint infections
